# Left Hepatic Lobe Agenesis With Ectopic Gallbladder

**DOI:** 10.7759/cureus.16131

**Published:** 2021-07-03

**Authors:** Raana Kanwal, Samina Akhtar

**Affiliations:** 1 Diagnostic Radiology, Shifa International Hospital, Islamabad, PAK

**Keywords:** ectopic gallbladder, left lobe agenesis, cholecystectomy, left portal vein, left hepatic vein

## Abstract

An ectopically located gallbladder is a rare entity. Here, we present a case of an ectopic gallbladder with left hepatic lobe agenesis. In this study, we describe the case of a 56-year-old male who was a known diabetic patient. He presented with abdominal pain, which started two weeks prior. Computed tomography (CT) abdomen with contrast was advised by the primary team, which showed an incidental ectopic gallbladder along the right posterior-inferior margins of the liver. Associated with it, there was complete agenesis of the left hepatic lobe, including absent segments II, III, and IV. Most of the commonly encountered ectopic positions include intrahepatic, transverse, retrohepatic, retroperitoneal, suprahepatic, falciform ligament, or under the left liver lobe. Ectopic gallbladders have clinical significance as they alter the clinical presentation of cholecystitis. They create technical challenges during cholecystectomy and other biliary operations and cause misdiagnosis in imaging. A thorough inspection of the biliary tract in patients undergoing surgery is suggested before electrocoagulation. A radiologist must always inform the clinician about the existence of an aberrant gallbladder.

## Introduction

Rarely reported congenital liver anomalies include hypoplasia or agenesis of hepatic lobes, absent segment, and agenesis of gallbladder [1]. Agenesis of the hepatic lobe is an incidental finding as the patient remains asymptomatic [2]. Imaging modality has a crucial role in determining the anatomy, including vascular anomalies, biliary duct details, and variations. Ideally, contrast-enhanced computed tomography (CT) or magnetic resonance imaging (MRI) are modalities of choice, with MRI being of higher yield without radiation exposure and contrast administration [3]. Initially, acquired causes of lobar absence are essential to exclude [4]. To the best of our knowledge and literature review, associated ectopic gallbladder with left hepatic lobe agenesis is rarely reported.

## Case presentation

**Figure 1:**
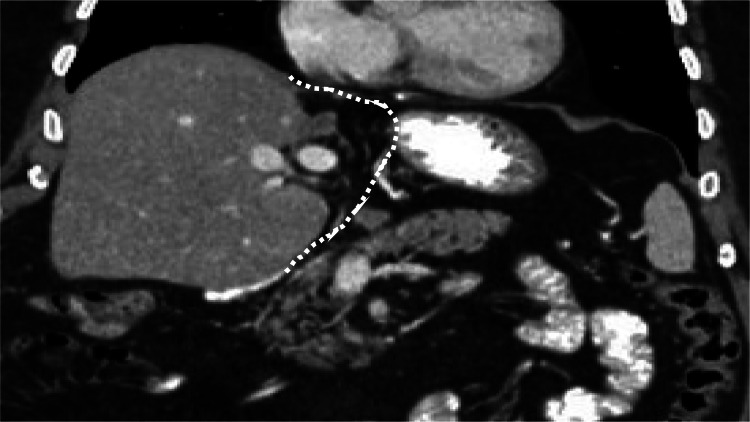
Complete agenesis of the left hepatic lobe, including segments II, III, and IV.

Here, we present a case of an ectopic gallbladder with left hepatic lobe agenesis. A 56-year-old male, who was a known diabetic patient, presented to the surgical outpatient department with a complaint of recurrent abdominal pain, which was persistent for the past two weeks. Contrast-enhanced CT abdomen and pelvis was acquired as a single-phase acquisition at approximately 60-90 seconds after intravenous contrast administration, which showed complete agenesis of the left hepatic lobe, including segments II, III, and IV (Figure 1).

**Figure 2:**
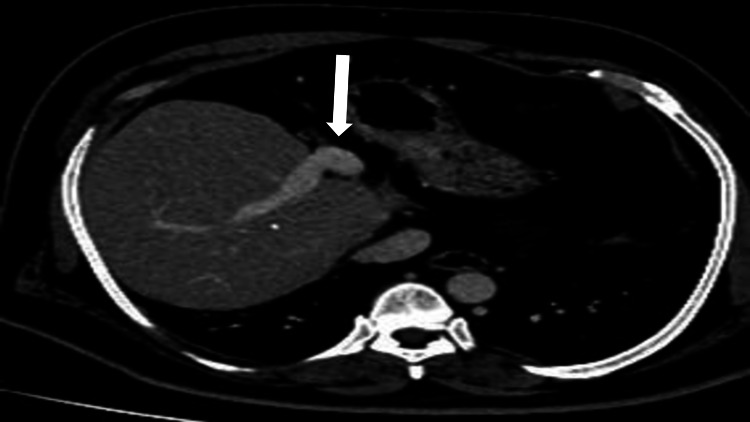
CT postcontrast axial view shows the main portal vein and its right branch. The left portal vein is not visualized.

The caudate lobe was normal. The main portal vein and its right branch were visualized, whereas the left portal vein was absent (Figure 2). Atrophic left hepatic vein was seen.

**Figure 3:**
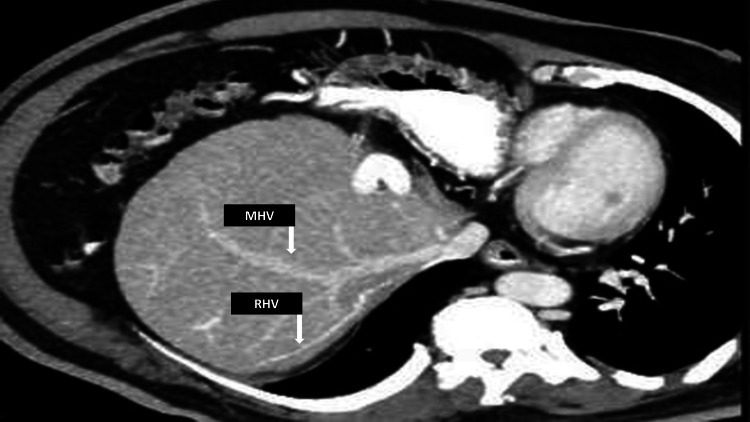
CT postcontrast axial view showing contrast opacified middle hepatic vein (MHV) and right hepatic vein (RHV).

While both middle and right hepatic veins were well-contrast filled, the hepatic artery was patent (Figure 3).

**Figure 4:**
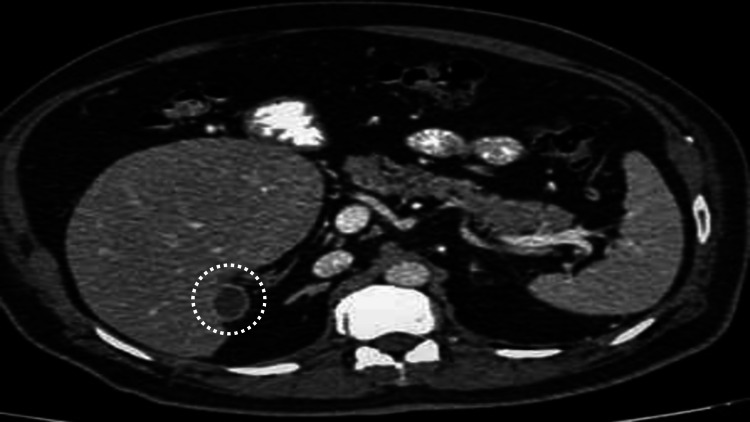
Gallbladder was found in an ectopic location along the posterior-inferior aspect of the liver.

Liver attenuation and enhancement were normal with smooth margins. No hepatic focal lesion was seen. No intra- or extrahepatic biliary dilatation was reported. The ectopic gallbladder was found incidentally along the right posterior-inferior margins of the liver (Figure 4).

**Figure 5:**
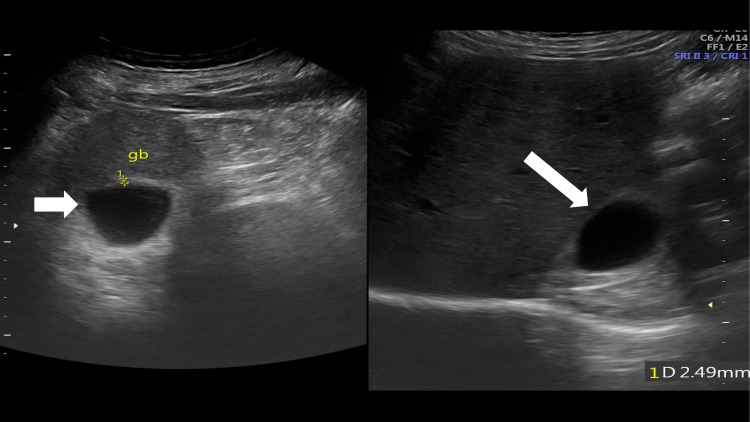
Ultrasound gray-scale images show an anechoic well-defined cystic structure in the posterior-inferior aspect of the liver, consistent with the gallbladder.

The patient did not have any history of abdominal surgery or trauma. Neither hepatic infection nor medication was reported in the medical history. Serum liver function tests were within the normal range. In the absence of known acquired causes of liver atrophy or presence of an associated visceral abnormality, the findings were attributed to be incidental and likely explain a congenital origin. Further dedicated ultrasound of liver and gallbladder was performed that confirmed the findings (Figure 5). 

The patient's follow-up investigation did not reveal any abdominal nor pelvic pathology. He was clinically diagnosed with gastroesophageal reflux disease (GERD). The patient was called and inquired about the medical treatment provided to him, to which he showed a satisfactory response.

## Discussion

Congenital liver anomalies are rarely reported. These include hypoplasia, deformed or agenesis of hepatic lobes, absent segment, and agenesis of the gallbladder [1]. Agenesis of the hepatic lobe is an incidental finding as the patient remains asymptomatic [2]. It was reported for the first time in 1932 by Arnold and Ashley-Montagu and was defined as a congenital disorder of the hepatic lobe. Imaging modality has a crucial role in determining the anatomy, including vascular anomalies, biliary duct details, and variations. Ideally, contrast-enhanced CT or MRI are modalities of choice, with MRI being of higher yield without radiation exposure and contrast administration [3]. Initially, acquired causes of lobar absence are essential to exclude, which may include traumatic, vascular, infectious, carcinomatous, or metabolic [4]. Specifically, left hepatic lobe agenesis may be due to multiple causes. This may be due to bile duct obstruction, portal vein obstruction, and bile duct dilatation having a mass effect on the left portal vein or severe malnutrition [5]. Similar to Chou et al., we also diagnosed liver lobe agenesis on a CT scan [6-8].

To the best of our knowledge and literature review, associated ectopic gallbladder with left hepatic lobe agenesis is rarely reported [9]. It is frequently reported with the right hepatic lobe, with isolated incidence reported to be only 0.007%-0.13% [10].

Unusual biliary symptoms require keen observation, where misinterpretation of imaging is crucial [11]. The first case of an ectopic gallbladder was reported by Bergmanin in 1702, with several cases reported later [12]. However, to our knowledge, gallbladder agenesis with associated left hepatic lobe is rare. The commonest site for ectopic gallbladder includes intrahepatic, retrohepatic, retroduodenal, retroperitoneal, retropancreatic, within the leaves of lesser omentum or within falciform ligament [13]. Preoperative vigilant observation for gallbladder location is essential as it might cause iatrogenic injury at the triangle of Callot [9]. Similar to liver anomalies, this anomaly too results from altering the natural course of embryogenesis.

Radiological imaging is helpful in knowing the presurgical anatomy. These may include computed tomography (CT), endoscopic ultrasound (EUS), and magnetic resonance cholangiopancreatography (MRCP). Alternative to invasive exploratory laparotomy or intraoperative cholangiography, radiological imaging has an added advantage of being non-invasive [14]. Multimodality imaging is helpful in making an accurate diagnosis [15]. Orientation to the correct diagnosis of these anomalies is crucial for radiologists, as they are the frontline person in avoiding misinterpretation. Therefore, a radiologist must know about this condition. This may help surgeons to avoid postoperative complications as well [16].

## Conclusions

Left hepatic lobe agenesis with retrohepatic gallbladder is a rare anomaly. It is challenging for both radiologists and surgeons. Both should be aware of this condition in surgical planning and for better patient management.
